# Human milk extracellular vesicles preserve bronchial epithelial barrier integrity and reduce TLR3‐induced inflammation *in vitro*

**DOI:** 10.1002/jex2.54

**Published:** 2022-08-31

**Authors:** Nikita Karra, Martijn J. C. Van Herwijnen, Marca H. M. Wauben, Emily Jane Swindle, Hywel Morgan

**Affiliations:** ^1^ Electronics and Computer Science, Faculty of Physical Sciences and Engineering University of Southampton Southampton UK; ^2^ Department of Biomolecular Health Sciences Faculty of Veterinary Medicine Utrecht University Utrecht Netherlands; ^3^ Clinical and Experimental Sciences Faculty of Medicine University of Southampton Southampton UK; ^4^ Institute for Life Sciences University of Southampton Southampton UK

**Keywords:** anti‐inflammatory, barrier integrity, bronchial epithelium, exosomes, extracellular vesicles, milk

## Abstract

Breast milk is essential for facilitating the growth and development of infants and for providing immune protection against viral infections in the infant's airways. Yet, regulation of inflammation by milk components may be needed to reduce immune pathology. While milk‐derived extracellular vesicles (EVs) are bestowed with immunomodulatory capacities, their role in bronchial epithelial barrier function and inflammation has not yet been examined. We hypothesised that during feeding, milk is not only ingested, but aerosols containing milk EVs are inhaled and locally delivered to the infant's airways to suppress aberrant inflammation. A bronchial epithelial model of viral infection was used to explore the direct effect of milk EVs on cellular barrier function and cytokine release during stimulation with a viral dsRNA analogue (Poly I:C). We demonstrate that milk EVs improved the dsRNA‐mediated decrease in ionic barrier integrity, limited tight junction reorganisation and reduced inflammatory cytokine production (IL‐6, IL‐8 and TNF‐α). This protective response was EV‐mediated, could be successfully titrated and exhibited a time‐dependent response. The results indicate that if EV‐containing milk aerosols are inhaled during feeding, this may lead to protection of the airway integrity from adverse inflammatory effects.

## 1 INTRODUCTION

The importance of breastfeeding is well established; it provides nutrition, facilitates growth and development, and protects the infant from infection whilst the adaptive immune system develops (Castellote et al., 2011; Galley & Besner, 2020). It also regulates inflammatory responses (Zonneveld et al., 2021) with a lower incidence of infectious morbidity and mortality in infants (Victora et al., 2016). Breast milk contains a multitude of components, such as milk fat globules, casein micelles, immune cells, oligosaccharides, microbes and EVs, each with their own function (Castellote et al., 2011; Van Herwijnen et al., 2016; Zonneveld et al., 2014). Milk EVs are nanosized lipid bilayer enclosed particles released by a multitude of cells, such as epithelial cells and immune cells (Van Herwijnen et al., 2016). Milk EVs can provide communication between mother and child, delivered via their cargo that includes proteins, lipids, small RNAs and metabolites (Galley & Besner, 2020; Zonneveld et al., 2021). Because of the oral route of breast milk, there has been a focus on milk EV function in the gastro‐intestinal tract. Milk EVs have been shown to dampen endosomal toll‐like receptor‐induced immune responses (TLR‐3, TLR‐9) and protect against oxidative stress injury in the gut (Dong et al., 2020; Gao et al., 2019; Miyake et al., 2019; Zonneveld et al., 2021). However, milk EVs may also play a role in reducing viral‐induced inflammation in the airways (Van Herwijnen et al., 2016) as milk aerosols are thought to be inhaled during breastfeeding, as infants use cyclical sucking and breathing patterns with intermittent windows to swallow milk (Goldfield et al., 2006). The existence of milk aerosols and their inhalation has been further demonstrated during occupational tasks, for example, milking cows and walking through milk storage rooms (Ramirez & Bahna, 2009). Since the airways have a large surface area that interacts with the external environment, colloidal material present in aerosols can be taken up, making the airways highly susceptible to damage or inflammation induced by these particulates, toxins, microorganisms or allergens (Karra et al., 2019). Impairment of epithelial barrier function and repeated infections in the first 3 years of life can significantly increase the risk of developing asthma (Holgate et al., 2009). Although there is a comprehensive insight into the anti‐inflammatory capabilities of EVs, a possible physiological role of milk EVs in controlling bronchial inflammation has not yet been investigated.

This study investigated the impact of milk EVs on a bronchial epithelial cell line when challenged in the presence or absence of a viral dsRNA analogue (Poly I:C), focusing on barrier integrity (determined from ionic permeability and immunofluorescent staining analysis) and cytokine release.

## 2 METHODS AND MATERIALS

### 2.1 EV isolation

Fresh and mature human milk was collected by three healthy mothers as previously described (Zonneveld et al., 2014) using an electric breast pump. Donors had a mean age of 33± 1 years, were at a mean lactational stage of 5.3 ± 1.5 months and had a mean parity of 1.7 ± 0.6. Informed consent was given by the donors and this study was approved by the local ethics committee. EVs and EV‐depleted (EV‐dpl) procedural controls were isolated as previously described (Zonneveld et al., 2021) using differential centrifugation, density gradient separation and size exclusion chromatography. Isolated EV and EV‐dpl samples were eluted in minimal essential medium MEM and Glutamax (Gibco) and stored (at ‐80°C) and shipped on dry ice until use. We previously validated this EV isolation protocol and characterized two out of three donor samples (milk EVs and EV‐dpl) used in this study by nanoparticle tracking analysis (NTA) and Western blot analysis (Zonneveld et al., 2021). Data are available in the EV‐TRACK knowledgebase (EV‐TRACK ID: EV200007) (Van Deun et al., 2017). Samples were freeze‐thawed once to aliquot into smaller volumes and supplemented with 1% Penicillin‐Streptomycin and 10% foetal bovine serum (FBS) (Life Technologies) and stored at ‐80°C until required.

### 2.2 Cell culture and challenge

The bronchial epithelial cell (BEC) line 16HBE14o‐ was used, owing to its ability to form tight junctions between cells leading to the generation of a polarised epithelial barrier with a high transepithelial electrical resistance (a measure of ionic permeability) (Callaghan et al., 2020). Cells were maintained in MEM and Glutamax (Gibco), supplemented with 1% Penicillin‐Streptomycin and 10% foetal bovine serum (FBS) (Life Technologies) termed complete MEM. Upon reaching ∼70% confluency, cells were incubated in Hanks' Balanced Salt Solution (HBSS) without Ca^2+^ and Mg^2+^ for 10 min at 37°C before detachment via a 5‐min incubation in 1 X trypsin (Life Technologies). The trypsin was then neutralised with complete MEM and the cell suspension pelleted at 300 *x g* for 5 min at 21°C. The cell pellet was resuspended in complete MEM and cells were counted using the trypan blue exclusion method. The cells were apically seeded onto collagen coated (30 μg/ml, Advanced Biomatrix) transwell inserts (Corning) at a density of 4.5×10^5^ cells/cm^2^ for 1 h to facilitate cell adhesion before measurements were conducted. Apical and basolateral media was replaced on days 2 and 4. Cells were apically challenged on day 5 following the formation of a barrier with 20 μl dsRNA analogue polyinosinic:polycytidylic acid (Poly I:C, 5 μg/ml) (Invivogen), with or without 5–100 μl of EV or EV‐dpl sample, using fresh complete MEM as a control. As cells were grown in transwells the total apical volume was 200 μl, EV samples were 1:1 diluted with apical media providing an EV concentration of half the physiological concentration found in breast milk for the highest volume used (100 μl), which was diluted further in the titration studies to generate 25%, 12.5%, 5% and 2.5% of the physiological concentration.

### 2.3 Trans Epithelial Resistance (TER)

Daily TER measurements were made using conventional “chop stick” electrodes and a Millicell ERS‐2 Voltohmmeter (Merck). Following the day 5 measurement, cells were challenged, and TER measurements made hourly for 5 h and then at 24 h.

### 2.4 ELISA

At the end of the experiment (24 hours after challenge), samples from the apical and basolateral compartments were centrifuged at 300 *x g* for 5 min. The resultant cell‐free supernatants were stored at ‐20°C until required. ELISAs were performed using the R&D Systems Duoset ELISA kits (IL‐8: DY208, IL‐6: DY206, TNF‐α: DY210) and accompanying Ancillary Kit (DY008). 96‐well plates were incubated with capture antibody (IL‐8: 4 μg/ml, IL‐6: 2 μg/ml, TNF‐α: 4 μg/ml) overnight at room temperature. Plates were washed thrice in wash buffer (0.05% Tween‐20 in PBS), and incubated with block buffer (1% BSA in PBS, pH 7.2‐7.4, 0.2 μm filtered) for IL‐8 or in reagent diluent (0.1% BSA 0.05% Tween‐20 in Tris Buffered Saline, pH 7.2‐7.4, 0.2 μm sterile filtered) for IL‐6 and TNF‐α for a minimum of 1 hour before being rinsed thrice in wash buffer. Experimental apical and basolateral supernatant samples were thawed on ice and diluted in reagent diluent using optimised dilutions outlined in the ESI and incubated with the standards for 2 hours in the dark at room temperature. The plate was washed again and incubated for 2 hours in the dark with a detection antibody (IL‐8: 10 ng/ml, IL‐6: 50 ng/ml and TNF‐α: 400 ng/ml) prior to rinsing (3X) and a 20‐minute incubation in the dark with HRP‐solution (1:40 dilution) at room temperature. The plate was then washed again (3X) and incubated with 1 X TMB (Thermo Fisher Scientific) at room temperature with periodic gentle agitation until a colour change was detected then the reaction is stopped with 2N Sulphuric Acid and the plate read at 450 nm and 570 nm following a 30 s shake at 400 rpm.

### 2.5 Immunofluorescence staining

Samples were fixed in 4% Paraformaldehyde (PFA) for 20 min and stored in PBS at 4°C. Samples were permeabilised with 0.1% Triton X‐100 in PBS and blocked with PBS plus 2% BSA and 0.1% Tween 20 and incubated overnight with Acti‐stain555‐phalloidin (Cytoskeleton) and AlexaFlour®488‐ conjugated anti‐human mouse occludin antibody (Life Technologies) at 4°C in a humidified chamber. Samples were washed thrice (PBS with 0.1% Tween‐20) and counterstained with DAPI nuclear stain before rinsing with PBS and 0.1% Tween‐20 solution (thrice) and dH_2_O. Samples were mounted onto glass coverslips using Mowiol (Merck) and were aligned using a Leica DMI 6000 inverted fluorescence microscope prior to images being captured at 63X using confocal microscopy in xyz mode (Leica TCS‐SP8 laser scanning microscope) using laser wavelengths of 405 nm (DAPI), 561 nm (Actin) and 488 nm (Occludin).

### 2.6 Data analysis

All results are presented as mean and standard deviation. Statistical analysis was performed using Graphpad Prism. A Shapiro‐Wilk test assessed the data for normality. A one‐way ANOVA with a Bonferroni post‐test or Friedman's Test with a Dunn's post‐test correction was used to calculate statistical significance. Results were considered statistically significant when *P* < 0.05 where *P≤0.05, **P≤0.01, ***P≤0.001, ****P≤0.0001.

## 3 RESULTS

### 3.1 Milk EVs preserve bronchial epithelial barrier integrity during inflammation

**FIGURE 1:**
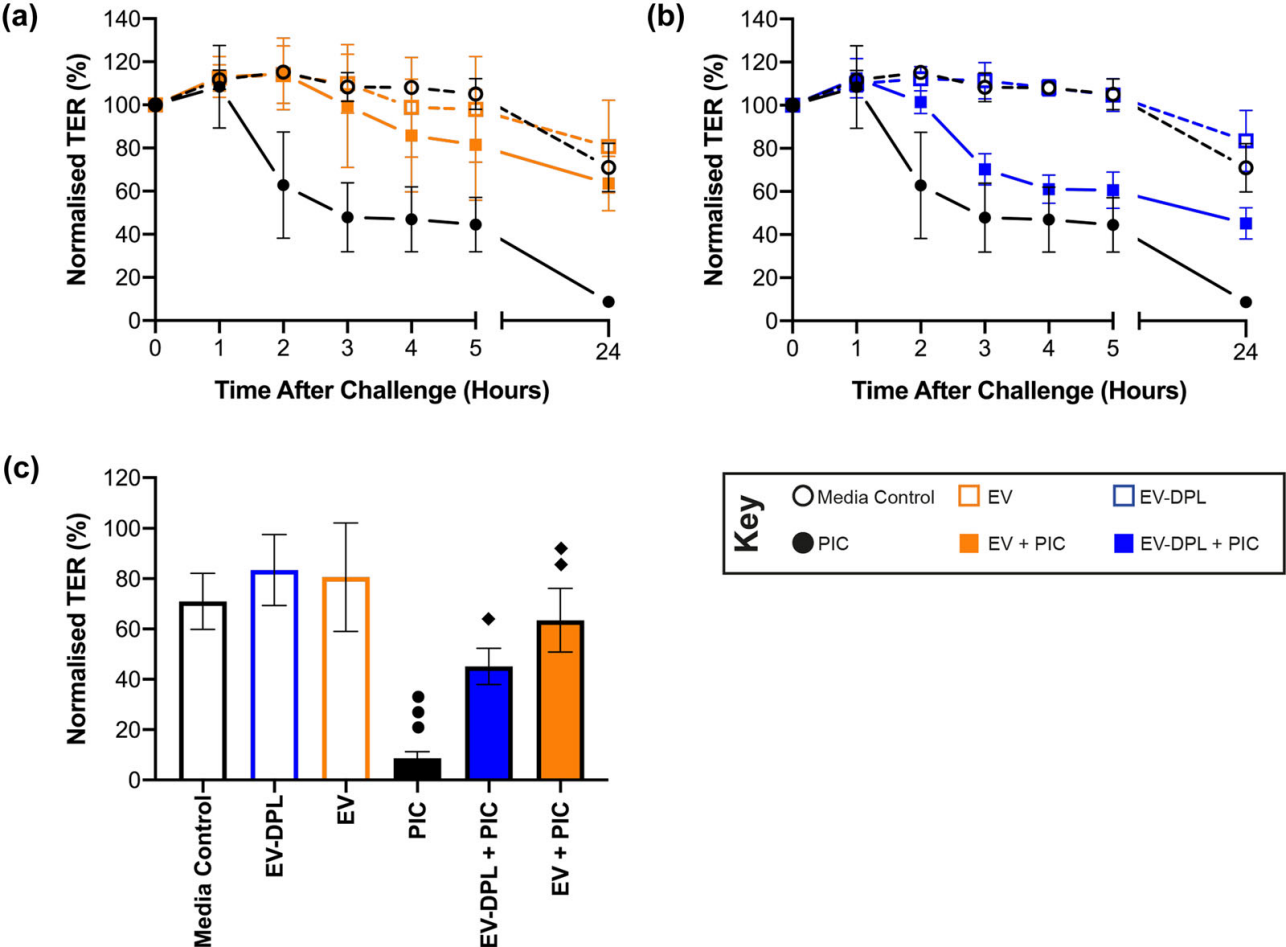
Bronchial epithelial barrier integrity as a function of time following challenge with Poly I:C and milk EVs. Polarised 16HBE14o‐ cells were apically challenged with Poly I:C (5 μg/ml) in the presence or absence (100 μl) of EV or EV‐DPL samples and the TER measured periodically over a 24‐h period. (a) is the EV data with control media and Poly I:C (PIC); (b) is the corresponding EV‐dpl data and (c) normalised TER values of the 24‐h time points. The TER is normalised to the value at day 5 (100%). Results are the average ± standard deviation of *n* = 3 repeats in triplicate (three milk donors in singlet). The different symbols detail the significance of multiple comparisons: ●●● *P*≤0.001 versus media control, ♦ *P*≤0.05 and ♦♦*P*≤0.01 versus Poly I:C as determined by one‐way ANOVA with Bonferroni correction for multiple testing

To determine the anti‐inflammatory capacity of milk EVs on BECs during viral induced inflammation in the airways, bronchial epithelial cells (16HBE14o‐) were apically challenged after 5 days of growth (ESI) with Poly I:C (a dsRNA analogue recognized by TLR3) in the presence and absence of EVs or EV‐depleted controls (EV‐dpl). The barrier response was monitored over 24 hours. For control cells without Poly I:C challenge (media control), the TER remained largely unchanged during the first 5 hours of measurement, declining gradually over 24 hours (Figure 1a). A similar trend in TER was observed when cells were exposed to milk EVs (Figure 1a) or the procedural EV‐dpl control (Figure 1b), indicating no detrimental or stimulatory effects of milk EVs. In contrast, exposure of cells to Poly I:C reduced the TER as expected by over 50% in the first 3 hours after challenge (Figure 1a,b). Remarkedly, the Poly I:C‐induced loss in TER could be overcome by the presence of milk EVs (Figure 1a), where the TER remained at approximately 100% over the first 5 hours, reducing at 24 hours similar to the media control. This contrasts with the Poly I:C‐induced loss in TER in the presence of EV‐dpl control (Figure 1b), where the TER already decreased in the first hours.

Figure 1(c) summarises these data in terms of mean (normalised) TER 24 hours post challenge. There was no significant difference between the media controls in the absence or presence of EV‐dpl control or milk EVs. This was in sharp contrast with the TER of cells challenged with Poly I:C alone which was significantly reduced at 24 hours. Addition of EVs to Poly I:C challenged cells reversed the decline in TER substantially, whilst EV‐dpl samples demonstrated subdued protection.

### 3.2 Milk EVs modulate inflammatory cytokine production during stimulation of BECs with Poly I:C

**FIGURE 2:**
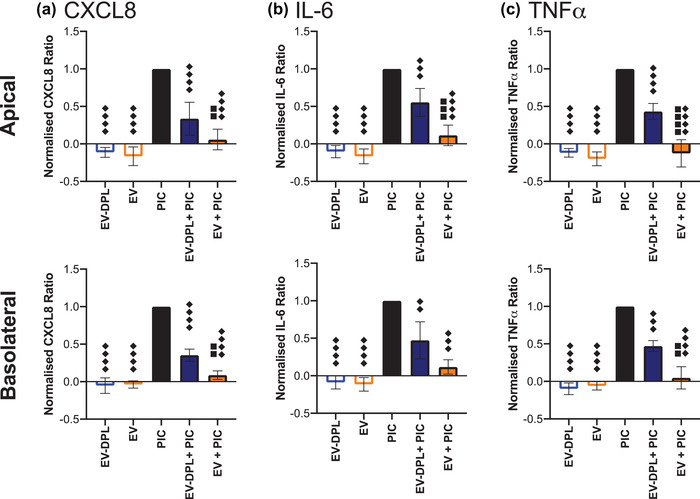
Milk EV modulation of cytokine response following challenge with Poly I:C. Polarised 16HBE14o‐ cells were apically challenged with Poly I:C (5 μg/ml) in the presence or absence (100 μl) of EV or EV‐DPL samples. 24 h after challenge the apical and basolateral samples were centrifuged at 300 x g for 5 min and the supernatants analysed for IL‐8, IL‐6 and TNF‐α by ELISA. The cytokine concentrations were plotted by first subtracting the unstimulated media concentrations then normalising to concentration after Poly I:C challenge. The different symbols detail the significance of multiple comparisons: ♦♦♦ *P*≤0.001 and ♦♦♦♦ *P*≤0.0001 versus Poly I:C and ■■ *P*≤0.01, ■■■ *P*≤0.001 and ■■■■ *P*≤0.0001 versus EV‐dpl + Poly I:C as determined by one‐way ANOVA with Bonferroni correction for multiple testing. The data shows the average ± standard deviation of *n* = 3 repeats in triplicate (three donors in singlet)

Following stimulation with Poly I:C, BECs produce pro‐inflammatory cytokines including IL‐6, IL‐8 and TNF‐α that initiate and perpetuate inflammatory response (Chow et al., 2010; Lever et al., 2015). The concentration of inflammatory cytokines (IL‐8, IL‐6 and TNF‐α) was measured 24 hours post challenge by ELISA and the results are summarised in Figure 2. To facilitate comparison of the data, the concentrations are normalised by subtracting the cytokine concentrations for the media control and normalising to the Poly I:C values.

Figure 2 shows that cells treated with EV or EV‐dpl samples only produced levels of IL‐8, IL‐6 and TNF‐α similar to the unstimulated media control. When cells were challenged with Poly I:C in the presence of EV‐dpl, cytokine release was reduced in both the apical and basolateral compartments by 64% and 65% for IL‐8, 45% and 53% for IL‐6 and 57% and 53% for TNF‐α, respectively. However, significantly stronger effects were observed for cells treated with EVs, with a substantial reduction of ∼90% in the cytokine ratios for IL‐8 and IL‐6, whilst TNF‐α release was inhibited completely in the apical compartment and significantly reduced by 95% in the basolateral compartment. These data suggest that in the presence of EVs there is a significant modulation of Poly I:C‐induced cytokine production, whilst a smaller effect is observed with EV‐dpl samples.

### 3.3 Extracellular vesicles display concentration dependent modulation of barrier integrity following challenge with dsRNA analogue

To determine whether the protective function of EVs was concentration dependent, different concentrations of EVs and EV‐dpl controls were tested in the presence of Poly I:C (5 μg/ml), by analysing the TER and IL‐8 release. The milk EV concentration was varied by diluting the EV samples in media, such that 100 μl of EVs in a 200 μl final volume equated to 50% of the physiological milk EV concentration down to 5 μl EVs in a final volume of 200 μl which equated to 2.5% of the physiological concentration.

**FIGURE 3:**
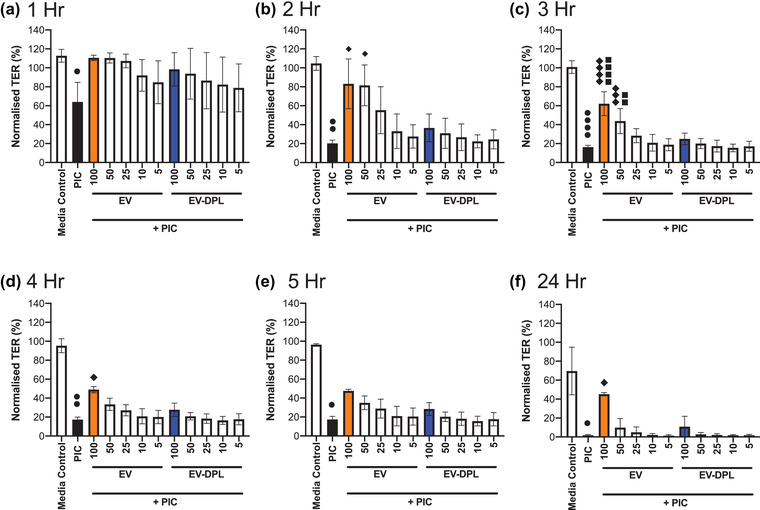
Concentration dependent modulation of TER with milk EVs in the presence of Poly I:C. Polarised 16HBE14o‐ cells were apically challenged with Poly I:C (5 μg/ml) in the presence or absence (5–100 μl) of EV or EV‐DPL samples to achieve a total apical volume of 200 μl. The TER was measured periodically over a 24‐hour period. Following apical challenge ionic barrier integrity was monitored by hourly transepithelial electrical resistance (TER) measurements for 5 hours (a) and then at 24 hours (b) using “chopstick” electrodes. The results in (A and B) are normalised to the day 5 value (100%). The different symbols detail the significance of multiple comparisons: ● *P*≤0.05, ●● *P*≤0.001 and ●●●● *P*≤0.0001 versus the Media Control, ♦ *P*≤0.05, ♦♦♦ *P*≤0.001 and ♦♦♦♦ *P*≤0.0001 versus Poly I:C and ■■ *P*≤0.01 and ■■■■ *P*≤0.0001 versus EV‐dpl + Poly I:C as determined by one‐way ANOVA or Friedman test with a Bonferroni or Dunn's correction for multiple testing. The data shows the average ± standard deviation of *n* = 3 repeats in triplicate (three donors in singlet)

Figure 3 shows the change in (normalised) TER over 24 hours for different concentrations of EVs following Poly I:C challenge. The barrier integrity was highly modulated in the presence of EV samples in a time and concentration‐dependent manner. One hour after challenge the higher volumes of EVs (25, 50 and 100 μl) generated TERs similar to the media control, whilst EV‐dpl samples also showed some modulation but to a lesser extent. The difference within concentration and sample type was more pronounced as time increased. After 2 hours, a protective effect of EVs was observed on the Poly I:C‐induced reduction in TER from 20% to 83% in the presence of 100 μl of EVs. After 3 hours a similar pattern was observed with EVs at 100 μl and 50 μl demonstrating a significant protection in the Poly I:C‐induced increase in ionic permeability of the epithelial barrier (from 16% for Poly I:C alone compared to 62% for 100 μl EVs + Poly I:C (Control = 100%)), (Figure 3c). While the effect was not as pronounced at 4–5 hours post stimulation, at 24 hours post challenge the highest concentration of EVs (100 μl) showed protection from the Poly I:C‐induced reduction in TER (from 2% for Poly I:C alone to 45% for EV + Poly I:C) while for the corresponding EV‐dpl control the TER dropped to 10% of the initial value. These data show that the effect of EVs on the TER is influenced by both time and concentration, where just 50% of physiological milk concentrations of EVs provide protection 24 hours after administration. Supporting this conclusion are the ELISA results where Poly I:C alone generated the greatest amount of IL‐8, which was markedly reduced in the presence of the 50% and 25% of the physiological concentration of EVs (100–50 μl) and EV‐dpl (100 μl) samples (see ESI).

### 3.4 Extracellular vesicles preserve tight junction organisation following challenge with Poly I:C

**FIGURE 4:**
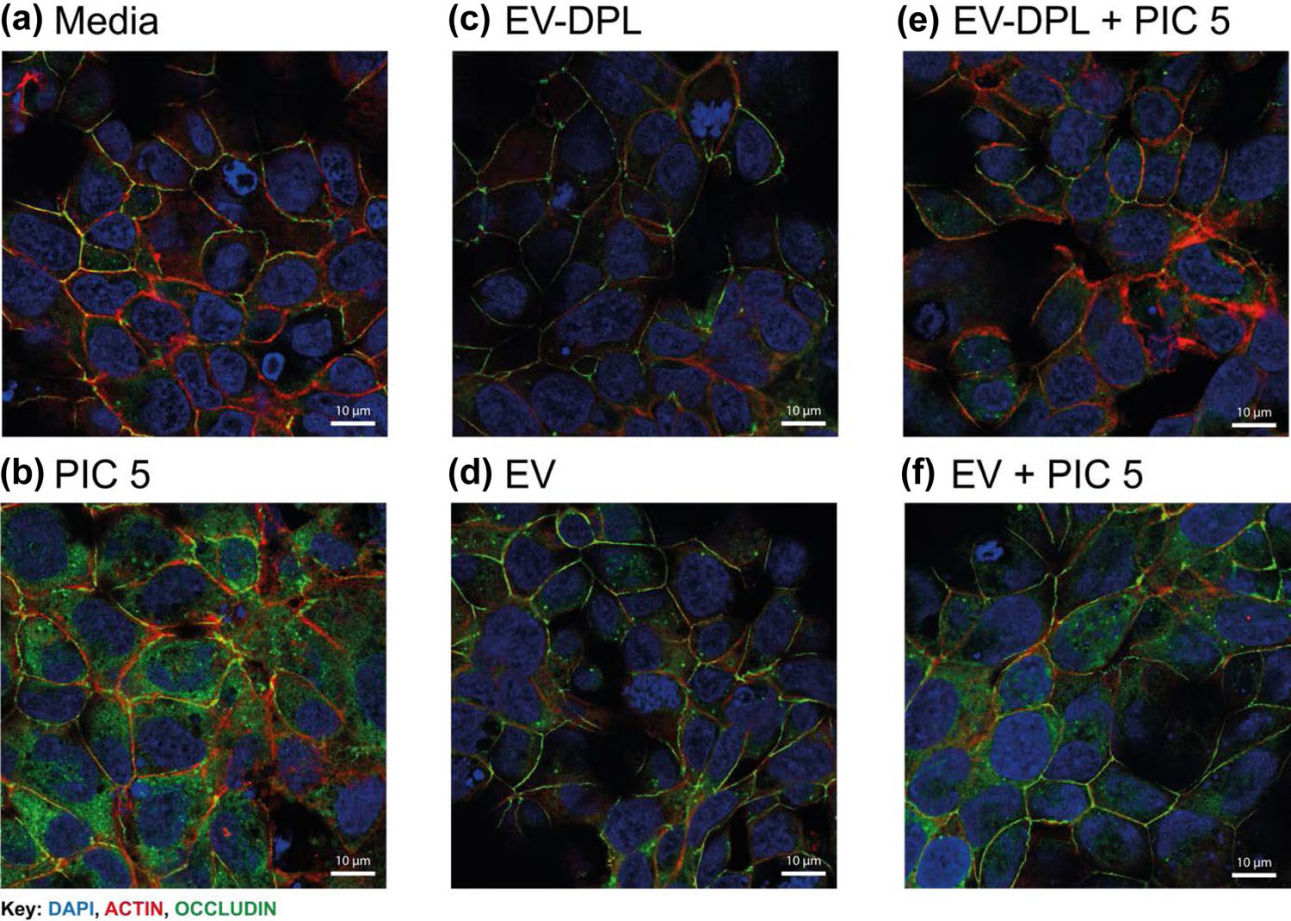
Bronchial epithelium tight junction organisation following challenge with Poly I:C in the absence of presence of milk EVs or EV‐dpl controls, imaged by immunofluorescent staining. Polarised 16HBE14o‐ cells were apically challenged with Poly I:C (5 μg/ml) in the presence or absence (100 μl) of EV or EV‐DPL samples. Samples were fixed using 4% PFA, 24‐h after challenge then immunofluorescently stained using DAPI nuclear, Actin cytoskeleton and occludin tight junction staining. Images show the occludin tight junction apicolateral region of Z projection stack only, captured using confocal imaging at 63X at wavelengths 405 (DAPI), 561 (Actin) and 488 (Occludin) (Leica TCS laser scanning microscope). The images are representative of *n* = 3 donors

To determine whether the EVs were able to maintain the integrity of tight junction organisation and formation, samples were immunofluorescently stained with an occludin antibody and counterstained with DAPI to visualise nuclei, and phalloidin to visualise the cytoskeleton. Overlaid images of tight junctions with nuclear and actin staining are shown in Figure 4 ESI. Media control, as well as EV‐dpl and EVs in the absence of Poly I:C had a similar apicolateral localisation of the tight junction protein occludin with pericellular location (Figure 4a, c and d). The distribution of occludin was disrupted in the presence of Poly I:C, where the occludin was re‐organised and concentrated at the cell‐cell junctions, also displaying gaps within the perimeter (Figure 4b). When EVs were applied in conjunction with Poly I:C, the tight junction distribution appeared similar to control conditions without Poly I:C with prominent staining encircling the cells, whereas the EV‐dpl samples were less able to modulate the Poly I:C response with weaker tight junction staining in comparison to EVs.

## 4 DISCUSSION

The airways host a large surface area with a continuous epithelium that interacts with the external environment (Leiva‐Juárez et al., 2018), where the barrier function is essential to maintain homeostasis. During the early stages of development before adaptive immunity has been gained, the infant is protected by the pattern recognition receptors (such as TLRs) expressed on airway epithelial cells, which are able to recognise pathogens and hazards and aid the development of adaptive immunity (Xu‐Chen et al., 2021). Breast milk plays an important role in this immunological programming, where it has been shown that in breast fed infants compared to formula fed infants a more anti‐inflammatory milieu is created (Kainonen et al., 2013). Breast milk is enriched in a variety of molecules that have demonstrated an ability to modulate immune responses (Thai & Gregory, 2020), an example of which are milk EVs that can dampen TLR activation in the oral cavity and gut (Dong et al., 2020; Gao et al., 2019; Miyake et al., 2019; Zonneveld et al., 2021). Since milk aerosols could be inhaled by infants during breast feeding due to the intermittent periods required to swallow milk during a continuous sucking and breathing cycle (Goldfield et al., 2006), this provides a route for milk particles to travel into the airways. To study possible immune modulatory effects of milk EVs on infant's airways we used the immortalised bronchial epithelial cell line 16HBE14o‐ (originally obtained from a 1 year old male (Callaghan et al., 2020)).

As indicated by the MISEV guidelines (Théry et al., 2018), in order to claim an EV‐mediated effect, appropriate controls should be used in experiments and effects should be titratable. For this, we deployed procedural controls made from EV‐depleted milk supernatant which was subjected to the same isolation protocol as the EV sample, resulting in a donor‐matched procedural milk control (Zonneveld et al., 2021). To evaluate potential immune modulatory effects of human milk EVs, we used a previously described method to test the effects of milk EVs at a physiological concentration (Zonneveld et al., 2021). In this volume‐based method, isolated EVs were tested in *in vitro* cell cultures at concentrations similar to their concentration in milk (9.3 × 10^10^ ± 1.2 × 10^10^ particles/ml) (Zonneveld et al., 2021). In our study cells were grown on Transwells at a liquid‐liquid interface and EV samples were 1:1 diluted with apical media giving an EV concentration at half the physiological concentration found in breast milk. This indicates that the observed effects in our study could be an underestimation of the physiological capacity to modulate epithelial cell responses. However, knowledge is lacking about the concentration of EVs in milk aerosols. By investigating the potency of different EV concentrations, we demonstrated that EV concentrations of 50% of the physiological concentration were able to inhibit viral dsRNA analogue (Poly I:C) activation of the immortalised bronchial epithelial cell line 16HBE14o‐up to 24 hours post‐challenge.

Poly I:C activates signalling via the toll like receptor 3 (TLR‐3) pathway (Théry et al., 2018) and has been shown to disassemble tight junction complexes and reorganise the cell actin cytoskeleton without inducing cell death (Blume et al., 2015; Rezaee et al., 2011). EVs were found to greatly circumvent Poly I:C induced barrier disruption compared to the control EV‐dpl samples. The addition of EVs allowed barrier maintenance comparable to the unstimulated condition (TER). This difference could be attributed in the variabilities observed in the organisation of tight junction occludin, as Poly I:C is able to reorganise and concentrate occludin to the cell‐cell junctions, generating gaps around the perimeter leading to a reduction in ionic barrier integrity (Blume et al., 2015; Rezaee et al., 2011). The addition of EVs rescued tight junctions, maintaining strong apicolateral staining around cells, whereas this ability was less pronounced in the EV‐dpl samples. These observations suggest that addition of EVs leads to modulation of the inflammatory response when cells are challenged with Poly I:C. The less pronounced modulatory effects of the EV‐dpl samples might be caused by residual EVs but also other bioactive agents present in human breast milk, such as human milk oligosaccharides that have shown in the intestines to modulate the TLR‐3 pathway, and lactoferrin that inhibits IL‐6, TNF‐α and IL‐8 production (Thai & Gregory, 2020). Our results demonstrate the need for a proper procedural matrix control when evaluating EV‐mediated effects.

The inflammatory cytokines IL‐6, IL‐8 and TNF‐α also play an important role in bronchial epithelial barrier function in terms of initiation and perpetuation of inflammatory responses (Chow et al., 2010), and addition of Poly I:C leads to an increase in their secretion (Lever et al., 2015). For the cytokine analysis performed in this study the cytokine concentrations were plotted in terms of relative change with respect to the values 24 hours after Poly I:C challenge. This approach reduced passage‐to‐passage variability and differences in the absolute secretion levels for each cytokine. Addition of Poly I:C induced high values of IL‐6 and IL‐8 secretion, but lower amounts of TNF‐α, as reported previously (Chow et al., 2010). The addition of EVs to the Poly I:C significantly inhibited the cytokine over‐expression by 80–90% for IL‐6 and IL‐8 and completely inhibited the Poly I:C mediated increase in TNF‐α expression, which was significantly less prominent in the EV‐dpl samples. These results were supported by previous findings for other cell types/organs where milk or stem cell EVs, respectively reduced IL‐6 production in a murine macrophage cell line (Ascanius et al., 2021) or in primary monocyte derived dendritic cells in response to LPS (Gomzikova et al., 2019; Reis et al., 2018). IL‐6 and TNF‐α were also reduced in a colitic murine model (Benmoussa et al., 2019) and in a hepatic murine cell line (Haga et al., 2017). Likewise, in an oral epithelial model, milk EVs were found to downregulate TLR‐3 activation via reduction of IL‐8 and IL‐6 expression (Zonneveld et al., 2021). Thus, milk EVs are able to significantly modulate the inflammatory response by actively reducing the expression of inflammatory cytokines.

## 5 CONCLUSION

This work demonstrates that milk EVs can modulate the TLR‐3 response (induced by Poly I:C) of bronchial epithelial cells *in vitro* by maintaining ionic barrier integrity and cellular organisation (occludin tight junctions and actin cytoskeleton). In addition, EVs reduce or inhibit apical and basolateral inflammatory cytokine production (IL‐6, IL‐8 and TNF‐α), in a concentration and time dependent manner. A better understanding of the physiological relationship between milk EVs and the airways is needed, as *in vivo* small milk particles could be inhaled during breastfeeding and provide a natural mechanism by which inflammation is regulated by breast milk. As such milk EVs might also provide the basis for new intranasal therapeutics. Future studies are required to further substantiate the physiological relevance.

## AUTHOR CONTRIBUTIONS

Nikita Karra: Writing ‐ Original Draft; Conceptualisation; Methodology; Formal Analysis; Investigation and Visualisation. Martijn van Herwijnen: Conceptualization; Methodology, Resources. Emily Jane Swindle: Conceptualization; Methodology; Formal Analysis; Resources; Supervision; Writing – review & editing. Marca Wauben: Conceptualization; Funding acquisition; Resources; Supervision; Writing ‐ review & editing. Hywel Morgan: Conceptualization; Funding acquisition; Resources; Supervision; Writing – review & editing.

## CONFLICT OF INTEREST

The authors declare no conflicts of interest.

## Supporting information

Supporting information.

## Data Availability

All data supporting this study is openly available from the University of Southampton repository at https://urldefense.com/v3/__, https://doi.org/10.5258/SOTON/D2180__;!!N11eV2iwtfs!vXf0TzbZ6i6IgvgvDyd5hhi_ePkhbUSSSpEaAvybDpaAReMpu4NlI31mmvK2BGzRT2q9jyNVo1rD$.
