# Supplementary material for: Human milk extracellular vesicles preserve bronchial epithelial barrier integrity and reduce TLR3‐induced inflammation in vitro
Source: J Extracell Biol. 2022 Aug 31;1(9):e54. doi: 10.1002/jex2.54 (PMC11080843; doi:10.1002/jex2.54)
Supplement: Supplementary file 1 — Supporting information. [file JEX2-1-e54-s001.docx]

**Human Milk Extracellular Vesicles Preserve Bronchial Epithelial Barrier Integrity**

**and Reduce TLR3-Induced Inflammation *in vitro***

Karra. N. ^a^ | Van Herwijnen.M.J.C ^b^ | Wauben. M.H.M ^b^ | Swindle. E.J. ^c,d^ | Morgan. H. ^a,d^

^a^ Electronics and Computer Science, Faculty of Physical Sciences and Engineering, ^c^ Clinical and Experimental Sciences, Faculty of Medicine, ^d^ Institute for Life Sciences,

University of Southampton. ^b^ Department of Biomolecular Health Sciences, Faculty of Veterinary Medicine, Utrecht University.

**Corresponding Author: Hywel Morgan (hm@ecs.soton.ac.uk)**

Electronic Supplementary Information

ESI 1: **Barrier integrity of bronchial epithelial cells after 5 days of growth.** 16HBE14o- cells were grown for 5 days with cell growth media changed in the apical and basolateral chambers on days 2 and 4. The TER was measured daily. Day 0 and Day 5 measurements for Figure 1 (A) and Figure 3 (B). Results are the average and standard deviation of n=3 independent experiments.

ESI 2: **Concentration dependent modulation of IL-8 release with and without extracellular vesicle immune protection when challenged with a viral dsRNA analogue**. Polarised 16HBE14o- cells were apically challenged with Poly I:C (5 μg/mL) in the presence or absence (5 to 100 μL) of EV or EV-DPL samples to achieve a total apical volume of 200 μL. 24 hours after challenge the apical (A) and basolateral (B) samples were centrifuged at 300 x g for 5 minutes with the supernatants analysed for IL-8 concentrations using ELISAs. The raw data was analysed as follows: the duplicates were averaged, the 520 nm reading subtracted from the 450 nm reading, and then the average blank was subtracted. The data was then extrapolated using GraphPad prism (Sigmoidal 4PL, X is concentration). The results show the average and standard deviation of n=3 repeats (3 donors in singlet). Statistical significance was determined using one-way ANOVA analysis with a Bonferroni post-test. Significance of results indicated as when p<0.05 = *. Symbols detail multiple comparisons: circle = Vs. Media Control, diamond for vs. Poly I:C and square for vs. EV-dpl + Poly I: C.

ESI 3: Dilution ratios for ELISA cytokine analysis.

|  | **Conditions without Poly I:C** | | **Conditions with Poly I:C** | |  |
| --- | --- | --- | --- | --- | --- |
|  | Apical | Basolateral | Apical | Basolateral | |
| IL-8 | 1 in 25 | 1 in 10 | 1 in 25 | 1 in 25 | |
| IL-6 | 1in 20 | 1 in 10 | 1 in 100 | 1 in 50 | |
| TNF-α | 1 in 2 | 1 in 2 | 1 in 5 | 1 in 5 | |
